# Analysis of the use of codon pairs in the HE gene of the ISA virus shows a correlation between bias in HPR codon-pair use and mortality rates caused by the virus

**DOI:** 10.1186/1743-422X-10-180

**Published:** 2013-06-06

**Authors:** Mario Tello, José Miguel Saavedra, Eugenio Spencer

**Affiliations:** 1 Centro de Biotecnología Acuícola, Facultad de Química y Biología, Universidad de Santiago de Chile, Alameda 3363, Santiago, Chile

## Abstract

**Background:**

Segment 6 of the ISA virus codes for hemoagglutinin-esterase (HE). This segment is highly variable, with more than 26 variants identified. The major variation is observed in what is called the high polymorphism region (HPR). The role of the different HPR zones in the viral cycle or evolution remains unknown. However viruses that present the HPR0 are avirulent, while viruses with important deletions in this region have been responsible for outbreaks with high mortality rates. In this work, using bioinformatic tools, we examined the influence of different HPRs on the adaptation of HE genes to the host translational machinery and the relationship to observed virulence*.*

**Methods:**

Translational efficiency of HE genes and their HPR were estimated analyzing codon-pair bias (CPB), adaptation to host codon use (codon adaptation index - CAI) and the adaptation to available tRNAs (tAI). These values were correlated with reported mortality for the respective ISA virus and the ΔG of RNA folding. tRNA abundance was inferred from tRNA gene numbers identified in the *Salmo salar* genome using tRNAScan-SE. Statistical correlation between data was performed using a non-parametric test.

**Results:**

We found that HPR0 contains zones with codon pairs of low frequency and low availability of tRNA with respect to salmon codon-pair usage, suggesting that HPR modifies HE translational efficiency. Although calculating tAI was impossible because one third of tRNAs (~60.000) were tRNA-ala, translational efficiency measured by CPB shows that as HPR size increases, the CPB value of the HE gene decreases (P = 2x10^-7^, ρ = −0.675, n = 63) and that these values correlate positively with the mortality rates caused by the virus (ρ = 0.829, P = 2x10^-7^, n = 11). The mortality associated with different virus isolates or their corresponding HPR sizes were not related with the ΔG of HPR RNA folding, suggesting that the secondary structure of HPR RNA does not modify virulence.

**Conclusions:**

Our results suggest that HPR size affects the efficiency of gene translation, which modulates the virulence of the virus by a mechanism similar to that observed in production of live attenuated vaccines through deoptimization of codon-pair usage.

## Background

The infectious salmon anemia virus (ISAV) is an orthomyxovirus, composed of eight segments. [1]. Two phylogenetic groups have been identified, the European and the North American, which probably diverged at the beginning of the last century [2,3]. Within each group the viruses present minimal genetic differences with the exception of certain regions of high variability localized in genes encoding for glycoprotein F [4] and HE. The region of hyper-variability in HE genes (HPR) is located between the transmembrane region and the globular domain with hemagglutinin-esterase activity [5,6]. The function of HPR within the viral cycle is unknown. However, the type of HPR in the virus has been associated with the degree of observed virulence. For example, the HPR0 is found in avirulent ISAV strains, while HPR7b is associated with ISAV outbreaks in Chile with high mortality rates [7-9]. The molecular ratios that explain the relationship between the HPR and observed virulence have not yet been identified. However, the HPR does not modify esterase or hemagglutininin activity [10].

Far from being silent, synonymous mutations affect diverse cellular processes, such as translation kinetics, mRNA structure and protein folding [11]. These mutations are distributed differently in the genes according to their levels of expression. Highly expressed genes tend to use preferentially the most abundant synonymous codons [12,13]. The relationship between bias in synonymous codon usage and gene expression seems to be the consequence of the synchrony observed between the frequency of codon usage and the abundance of tRNA that recognizes it [12,14-19].

As well as the bias in codon usage, there is a bias in codon-pair usage, which is characteristic for each organism and independent of the frequency of codon usage [20]. As with the synonymous codons, the codon pairs are distributed in the genes differently according to their level of gene expression [21].

The ribosome traffic jam in the mRNA has been suggested as a common mechanism that explains the reduction in translation efficiency by introducing codons with low tRNA availability, or tRNAs with little compatibility among themselves, as well as the negative relationship between the formation of stable structures in the mRNA and the level of protein expression [22-25].

There are few examples among viruses of the influence of codon use on the viral cycle. Studies about the adaptation to host codon usage indicate that viral genes that codify for critical proteins tend to use the synonymous codons most represented in the host genome [26]. In Influenza A, the structure acquired by the 5’end of the codifying zone is fundamental for viral packaging [27,28]. In the hepatitis A virus, the presence of underrepresented codons in the host genome seems to be a strategy to avoid competition with cellular mRNAs and to increase adequate folding of the capsid protein [29,30]. In the influenza A and polio viruses, the introduction of synonymous codon pairs in the viral genome with a negative bias produces viable attenuated viral particles with reduced rates of protein synthesis and replication [31-33].

It has been suggested that the different HPR are probably the result of in-frame deletions from sectors of HPR0 [6,34]. These deletions could modify translation efficiency by elimination of underrepresented codons in a similar but opposite mechanism to what is observed in the generation in vitro of live attenuated vaccines by deoptimization of their codon-pair usage. In this work we analyze this hypothesis of the effect of HPR on the adaptation to host codon usage, tRNA availability, host codon-pair usage bias and the folding energy of the HE gene.

## Results

### The effect of the HPR on translation efficiency

Translation efficiency is governed by secondary structure of the mRNA [35] and by its adaptation to the tRNA pool [12]. To evaluate if the HPR affects virulence through the possible formation of a stable secondary structure that reduces the advance of ribosome, thus causing a decrease in the HE translation rate, we determined if there is a relationship between the HPR folding energy and observed virulence. Our results indicate the absence of a statistically significant relationship between folding ΔG and the size of the HPR region or associated mortality. However, the HPR0 region showed the most favorable energy (ΔG = −24.2 Kcal/mol) (Additional file 1: Figure S1).

The effect of the presence of the different HPRs on translation efficiency can also be evaluated by analyzing the degree of adaptation to the tRNA pool through the use of the tAI value, which corresponds to the geometric mean of the relative abundance of tRNA in the cells that recognize the codons present in the gene. The relative abundance of the tRNA can be calculated based on the number of copies of each tRNA present in the genome [12]. Using the tRNAScan-SE program, we identified 63857 genes in the *Salmo salar* genome that codify for tRNAs. Approximately 21085 of these genes codify for pseudogenes and another 20546 codify for alanine tRNA. Of the rest of the tRNAs, their abundances range from 2724 for methionine tRNA to 236 for tryptophan tRNA. As well, 353 untyped tRNA were identified and 20 genes for tRNA suppressors (Additional file 2: Table S1). As in the calculation of tAI, the relative abundance of the tRNAs that recognize the codons in the gene was normalized with respect to the codon with greater availability of tRNAs. The extreme disproportion between Ala-tRNA copies and the other tRNA for the other amino acids makes interpretation of the data problematic and could lead to erroneous conclusions. To avoid this problem, we continue the study using adaptation to host codon use and bias in codon-pair usage.

### Adaptation to host codon usage and host codon pair bias of the ISAV hemagglutinin-esterase gene

The codon adaptation index (CAI) is a good indicator of the adaptation to the host codon usage and a good predictor of translation efficiency [13]. Previous studies have indicated that ISAV is the least adapted orthomyxovirus to it host’s genome, sharing CAI values with less than 3% of the genes of *Salmo salar* (Tello, unpublished). Among these, the North American virus presents the lowest level of adaptation. If the HPR modifies translation efficiency through the accumulation of high or low frequency codons, it can be expected that the CAI values of hemagglutinin genes are proportional to the size of the HPR and the protein.

**Figure 1:**
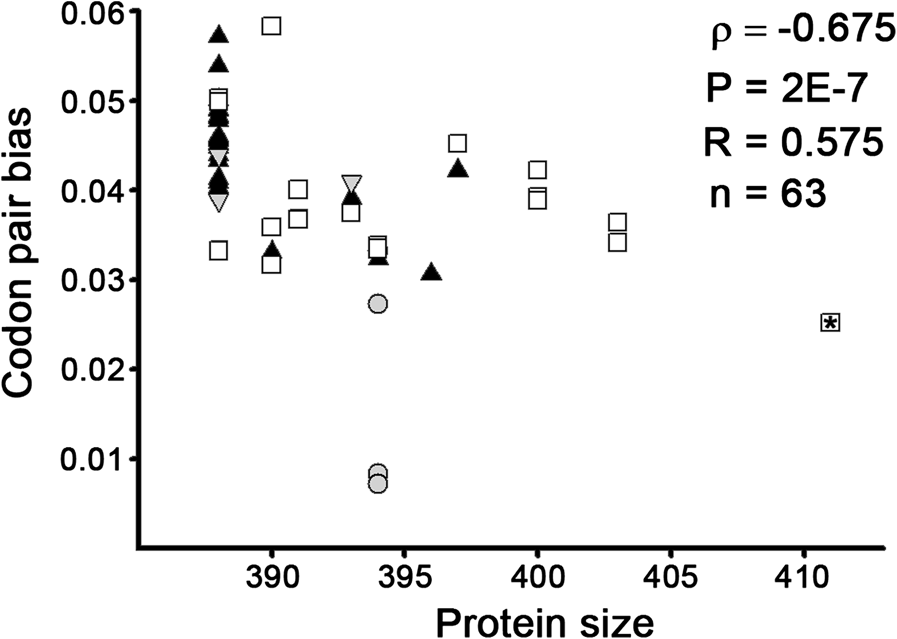
**Relationship between CPB and HE size.** The figure shows the CPB values of 63 proteins with complete sequences in the encoding region of the HE gene. The CPB values are shown for HE genes in the Norwegian virus (white squares), North American virus (gray circles), the Chilean virus (black triangles) and the British virus (inverted gray triangles). The square with a star indicates the HE gene of the HPR0 genotype.

Among the 63 analyzed sequences, no statistically significant relationships (P < 0.05) were found between the CAI value of the HE gene and the size of the proteins or observed virulence, but a relationship was observed between the CAI value of the HPR and the size of this region (ρ=0.393, P=0.002, n=63). As the translation efficiency is affected by the presence of underrepresented codon pair usage in the genome, we analyzed bias in the codon pair usage of the HE genes (Figure 1). The results show that HE shares CPB values with 20% of the salmon genes (Additional file 1: Figures S2 and S3). A statistically significant relationship was identified (ρ=−0.675, P=2x10^-7^, n=63) between CPB value and HE size. Of the 63 analyzed sequences, 3 were genes of Canadian viruses, which presented low CPB values. Among the viruses classified as European, the gene containing HPR0 had the lowest CPB value (Figure 1). This correlation increases when analyzing the relationship between the CPB value of the HPR region of the European isolates and the size of HPR region (ρ=−0.757, P=2x10^-7^, n=60).

When viruses were compared according to their country of isolation, there were significant differences (*t*-test, P = 0.008) in the mean CPB values of the HE gene of the Norwegian (CPB = 0.0394±0.007) and Chilean viruses (CPB = 0.0446±0.006), with the Chilean isolates presenting the higher CPB values (Additional file 1: Figure S4). When the CPB values of the HE gene are classified according to their HPR type, we note that the lowest CPB values are found in the HE genes containing HPR21 and HPR0, while the highest values are found in the HE genes containing HPR1, HPR15 and HRP7 (Additional file 1: Figure S5).

**Figure 2:**
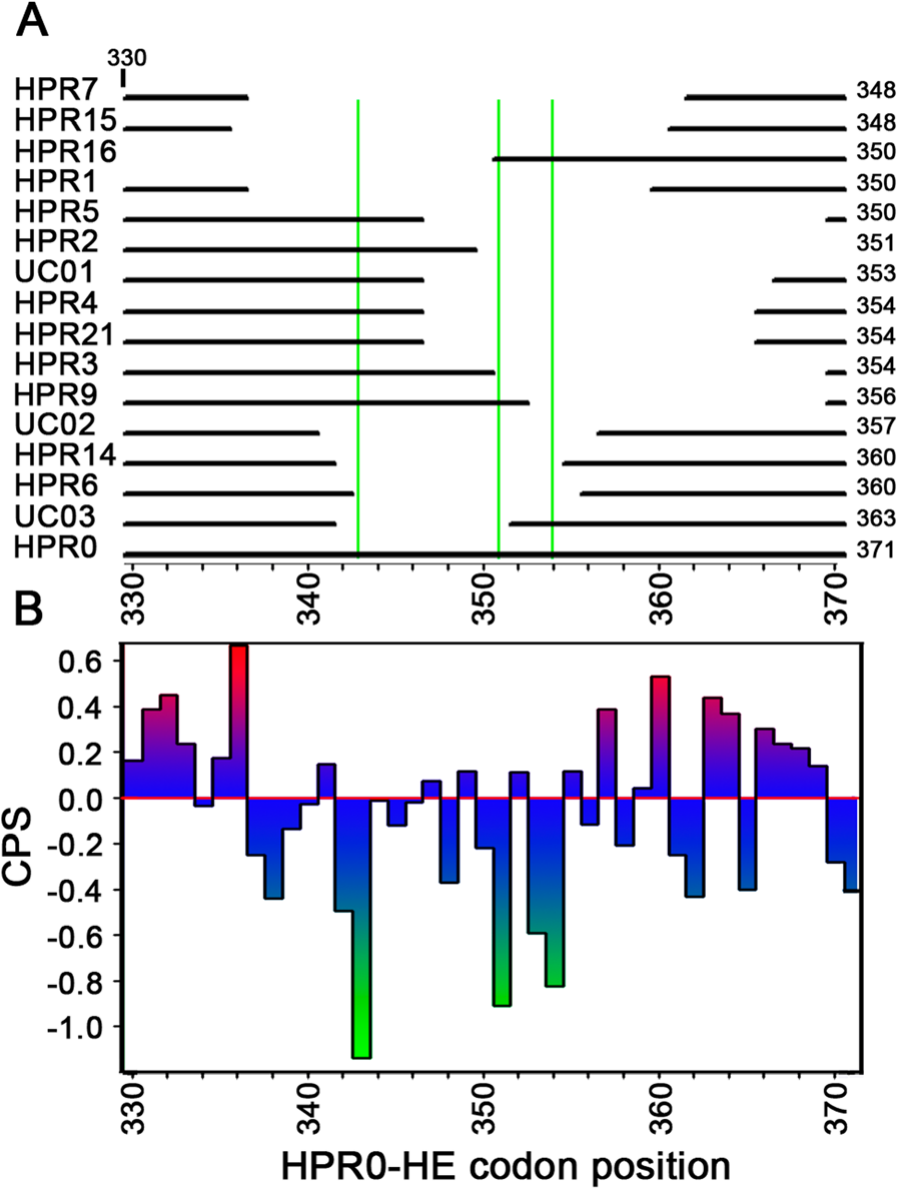
**Codon-pair score in the HPR region.** The figure shows an alignment scheme among the different HPR in the analyzed HE (Panel **A**). Panel **B** shows the codon-pair score (CPS) of the codon pairs in the HPR region of the ISAV HE-HPR0 gene. The green lines identify the codon pairs with lower CPS.

**Figure 3:**
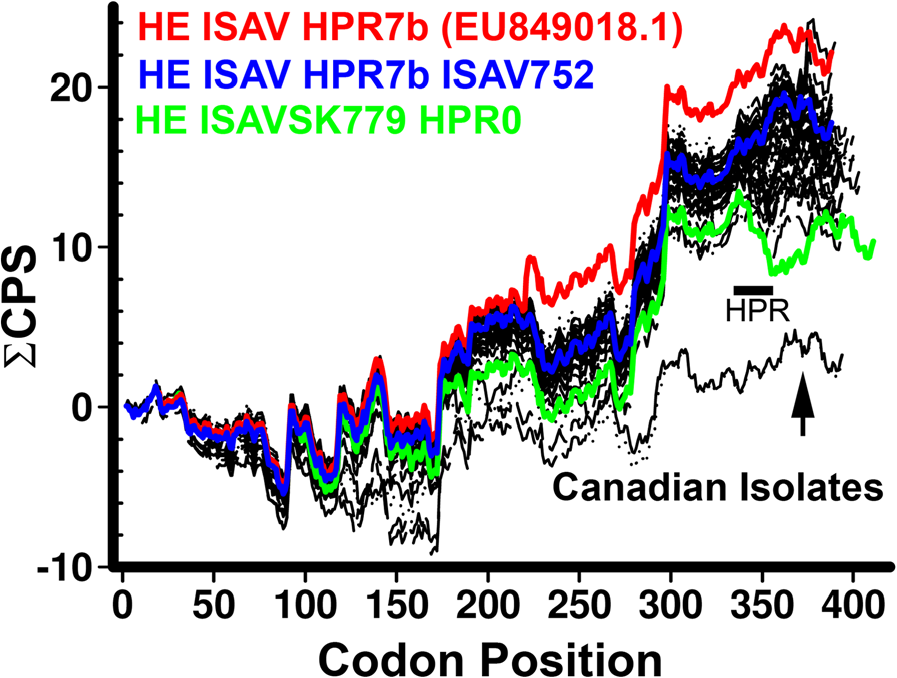
**Profile of CPS throughout the HE gene.** The figure shows the profile of accumulated CPS values for the 63 HE genes. We highlight the Chilean isolates with HPR 7b, the Canadian isolates and the HPR0 region.

To assess the impact of the HPR region on the total CPB value of HE, we determined the QCPB value. The results of this analysis show that the HPR0, HPR9, HPR3, HPR16, HPR2, HPR5, HPR4, UC010, UC011 and HPR6 regions negatively affect the CPB value of the gene that carries them, while HPR7, HPR1, HPR14, HPR15, UC04 and HPR21 increase total CPB value (Additional file 1: Figure S6). With the exception of HPR9 and HPR3, the HPR0 segment has the lowest CPB value, while HPR15, HPR7 and HPR1 present the highest CPB values (Additional file 1: Figure S7). The HPR0 and HPR9 regions had the most negative effect on total CPB value. Because the HPR0s had one of the lowest CPB values and most negatively affected total CPB value, we analyzed this region to find zones rich in codon pairs that are underrepresented in *Salmo salar*. This analysis shows that there are three points in the 343–344, 351–352 and 354–355 residues in the HPR0 region that present CPS values < 0.8, that is, that these codon pairs are underrepresented by at least two times. Most of the points with low CPB values are absent in the rest of the HPR variants (Figure 2). Comparison among the CPS profiles of the different genes shows that the most significant difference in the carboxyl terminal profiles are found in the HPR region. The viral isolates in Chile with the HPR7b show profiles with greater magnitude, in contrast to what is observed in the North American viruses and ISAV SK779 (HPR0). In the gene with HPR0, the HPR zone presents a descending profile that breaks an ascending ramp of CPB values present in the HPR variant with important deletions in the HPR zone (Figure 3).

### Relationship among codon-pair bias, HPR type and virulence

Our analysis consistently shows that the genes containing the HPR0 and HPR7 regions have extreme CPB values. The two HPRs are associated with opposite phenotypes: the HPR0 region is found in avirulent strains of ISAV, while the HPR7 region has been systematically found in Chilean outbreaks that have been responsible for eliminating 90% of salmon in production centers. To determine if there is a relationship among virulence, HPR type and codon-pair bias, we correlated our CPB values to the mortality rates reported by Mjeeland [36].

**Figure 4:**
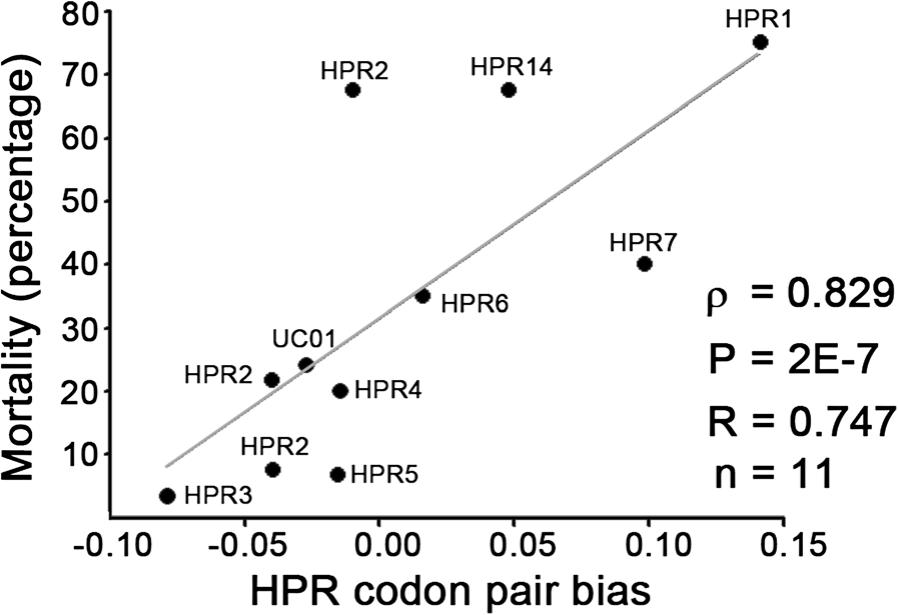
**Relationship between virulence and codon-pair bias (CPB).** The figure shows the relationship between mortality of ISAV1 and ISAV11 reported by Mjaaland, and the CPB values reached by their HPR regions. The statistical validity of the correlation was determined by the non-parametric Spearman rank order test.

These mortality rates constitute valuable information given that the virulence of 11 isolates of the ISA virus with different HPRs were analyzed and compared. The results of this correlation show that the CPB values of the HE gene correlate positively with mortality by direct injection (ρ = 0.597, P = 0.0467, n = 11) and with mortality rates with fish-to-fish transmission (TG2, ρ = 0.633, P=0.032, n=11). When we use only the CPB value of the HPR region, the correlation with mortality of the cohabitants (fish inoculated with the virus by injection) increases (ρ = 0.829, P = 2x10^-7^, n = 11) (Figure 4).

Extrapolation of mortality based on the CPB value of the HPR shows that the Norwegian viruses with HPR15 (AF378180.2 and AF302799.1) and the Chilean viruses with HPR1C (GU830908.1 and FJ594285.1) have an estimated mortality > 80%, which is higher than values observed in ISAV2 with HPR01. When we analyzed the CPB of the complete HE gene, we observed that most of the Chilean viruses have values between those of ISAV1 (HPR14) and ISAV2 (HPR01). The CPB value of the HE gene from Chilean isolates with HRP7b has a value of 0.122, which by extrapolation becomes a virus with similar characteristics to HPR1 (CPB = 0.146) in the isolates of ISAV2. ISAV2 has the highest mortality rate by ISAV injection (75%) and the second highest rate by cohabitation (28.9%), followed by the HPR14 in the ISAV1 isolate (46.1%) (Additional file 3: Table S2).

## Discussion

The results presented in this work indicate the existence of a relationship between bias in the use of codon pairs and the size of the HPR region or the HE gene. In particular, our analysis shows that the different types of HPR tend to eliminate regions that present underrepresented codon pairs (negative CPS index). In Influenza A and polio, the introduction of codon pairs with low CPS values is an effective way to reduce translation of the viral gene, reducing the production of viral particles and virulence. In contrast, the introduction of codon pairs with positive CPS increases protein expression [31,32]. The molecular explanation for this relationship is not clear, but it is probably because not all tRNA are compatible among themselves upon simultaneously occupying the A and P ribosome sites, as multivariate analysis suggests [37].

A universal codon distribution pattern has recently been described. In general the genes tend to accumulate codons poorly adapted to their tRNA pool among the first 50 codons, while the more adapted codons are found among the last 150 to 300 nucleotides [22]. It has been suggested that the presence of this adaptation ramp to the tRNA pool serves to avoid the ribosome traffic jam in the mRNA, the translation efficiency being inverse to the quantity of ribosomes in the mRNA. Thus, the presence of underrepresented codons could reduce translational efficiency and the advance and turn-over of ribosomes [22-24].

The HPR region is located at approximately 80 codons from the carboxyl terminal with respect to the gene with HPR0. According to the ramp observed in eukaryotic [22], this region should contain codons more adapted to the tRNA pool. The presence of underrepresented codon pairs in the HPR zone could decrease the velocity of the advance of ribosomes by the incompatibility among tRNA, being functionally equivalent to the use of codons with low levels of available tRNA. On the other hand, the elimination of these codon pairs could increase translation efficiency by decreasing the ribosome traffic jam in this region. The Chilean isolates with HPR7b present a ΣCPS profile with high CPS values and a continuous ramp toward the carboxyl terminal (Figure 3), which is interrupted in the virus with HPR0. Consistent with this, viruses containing HPR0 are associated with avirulent ISAV strains. On the other hand, HPR regions where codon pairs with negative CPS have been eliminated are associated with outbreaks with high mortality rates, such as HPR1 or HPR7b found in 80% of the pathogenic outbreaks in Chile [38,39]. The virulence values extrapolated for the Chilean isolates are consistent with the effect of these viruses on the Chilean salmon industry.

Current efforts to cultivate the virus in vitro with the HPR0 genotype have not been fruitful, which is why it remains unknown if these viruses have lower HE protein synthesis rates than viruses with the HPR7b genotype. Nevertheless, the correlations between the CPB values in the HE gene with the size of the region and the degree of pathogenicity of the ISA virus are consistent with the effect of HPR on translation efficiency.

Both immune escape due to antigenic variation produced by different HPRs [40] and modification of hemagglutinin-esterase activity can act as selection forces for the different HPR described. Recent experiments have determined that HPR does not modify hemagglutinin-esterase activity [10], suggesting that the biological activity of the protein does not explain the difference in observed virulence among ISAV isolates. There are variations in the protein sequence of the glycoproteins of influenza A that give rise to antigenic drift [41]. However, in influenza A, the variations occur by specific mutations that produce variations in the epitopes, in contrast to what occurs in ISAV, where the greatest variation observed in the HPR occurs by partial deletions in the HPR0 [34]. In ISAV, lesser variations have been observed on the surface of the HE protein [39]. However, it is not known if these mutations have an important effect on antigen-antibody recognition. Although antigenic variation in the ISAV hemagglutinin-esterase could act as a selective force, in our opinion this concept makes more sense in populations whose individuals are long-lived and acquire immunity after surviving a viral infection or vaccination. Unlike humans, salmon are short-lived (four years) and suffer outbreaks of ISAV with high levels of mortality. Moreover, variants in the HPR region appeared prior to the development of vaccines. Determining the degree of adaptation to the tRNA pool could help in confirming that the codons in the HPR region reduce translation efficiency. However, the estimation of the number of tRNA copies in *Salmo salar*, using the tRNAScan-SE program (which recognizes close to 100% of the tRNA in a genome), showed that *Salmo salar* has a large number of tRNA genes (>65000), which is much higher than the approximately 500 copies in humans and is on the order of the number found in other teleosts, like zebra fish (~45,000) [42]. Interestingly, close to a third of the identified tRNA is tRNA-Ala and another third is pseudogenes. This disproportionately high level of tRNA suggests the presence of a repeated SINE-type element, which presents homologous regions to those of the tRNA [43]. Repeated SINE-type elements have been described in salmonoids and other teleosts, but with leucine and lysine tRNA [44-47]. This suggests the presence of a new SINE-type in *Salmo salar*, although further studies will be necessary once the *S. salar* genome has been completely annotated to characterize this type of element. Despite the significant disproportion of Ala-tRNA compared to the other tRNAs, the W values of the codons in the HPR0 region show three areas, close to the area with negative CPS, where codons with low tRNA availability accumulate (Additional file 4: Table S3, Additional file 5: Table S4 and Additional file 1: Figure S8). This reinforces the idea that HPR0 is a zone of reduced translation efficiency.

In summary, our analysis shows a correlation between the CPB value of the HE gene or the HPR region and the virulence of the ISA virus. To date, we have not determined if the CPB value in the ISAV is related to gene translation. However, in vitro data from influenza A and the poliovirus strongly suggest the existence of this relationship. Although it is an over-simplification of the pathogenesis to explain virulence based solely on the adaptation of viral genes to the host’s codon pair usage, to our knowledge the correlations described in this work constitute the only natural example of de-optimization of codon pairs as the element correlating to the level of pathogenicity of a virus and constitute a reliable explanation of the role of the HPR region in the ISA virus as a modulating factor of virulence. Without doubt, the development of a reverse genetic technique could verify this relationship, but until this technique becomes available, sequence analysis is a useful tool.

## Materials and methods

### Calculation of tAI

We calculated tAI in accordance with what was established by dos Reis [12] (Equation 1 and 2), estimating the relative abundance of the tRNAs in function of the number of copies of each tRNA in the *Salmo salar* genome. The number of copies of each tRNA was determined with the tRNASCAN-SE program [48] in Linux, which was used to analyze the 555960 contigs in the GenBank database (Project ID: 72713) corresponding to the recently released salmon genome.

(1) $$W_{i}=\sum_{j=1}^{n_{i}}\left(1-S_{i}{}_{j}\right)\mathrm{tGN}C_{\mathrm{ij}}$$

where W_i_ is the relative adaptiveness of the codon i^th^ to the tRNA pool, n_i_ is the number of tRNA isoacceptors that recognize the i^th^ codon, tGCN_ij_ is the number of copies of the j^th^ tRNA gene that recognize the i^th^ codon, and S_ij_ is the selective constraint in the efficiency of codon-anticodon pairing.

(2) $$\mathrm{tA}I_{g}={\left(\prod_{k=1}^{l_{g}}w_{\mathrm{ikg}}\right)}^{1/l_{g}}$$

The adaptation of a gene to the tRNA pool is calculated according to equation 2, in which w_i_ is defined as the quotient between W_i_ and W_max_ (W_i_/W_max_), ikg is the codon defined by the k^th^ triplet of gene g, and l_g_ is the length of gene g in codifying codons.

### Determination of bias in codon-pair usage

Bias in codon-pair usage was determined according to what was described by Coleman [31]. To do this, we recalculated the codon-usage tables in Kazusa (http://www.kazusa.or.jp/codon/), using the information in the codifying regions of the *Salmo salar* genes available in Genebank. The sequence of the codifying regions of these genes was downloaded from the RefSeq database and subsequently filtered to eliminate redundant genes or genes with ambiguous nucleotides or with incomplete codifying regions. A total of 3485 sequences passed through the filters, which represented the entrance of 1168431 codons. Based on this set of sequences (Additional file 6: Table S5), we calculated the codon and codon-pair frequency, using scripts in the language Python. The observed frequency of amino acid pairs was deduced based on the sum of all the codon pairs that codify for the same combination of amino acids. Subsequently, we used these frequencies to calculate the CPS index (codon-pair score) using the equation described by Mueller et al. 2006 [33] (Equation 3), where f(AB) corresponds to the frequency of the appearance of the AB codon pair expressed in parts-per-thousands with respect to the total number of codon pairs in the genome. f(A) and f(B) correspond to the abundance of each of the individual codons expressed in parts-per-thousands with respect to the total number of codons in the genome. f(X) and f(Y) corresponds to the amino acids codified by the A and B codons respectively and f(XY) is the abundance of the amino acid pairs in all the proteins of the organism under study. The CPS values of the 3271 codon pairs in *Salmo salar* can be found in the supplementary material (Additional file 7: Table S6). We calculated the bias in codon usage as the arithmetic mean of the codon-pair score of a gene (CPS) described by Coleman et al. (2006) Equation 4, in which i is the *i*^*th*^ codon pair of a gene and *l* is the total length of the gene expressed in the quantity of codified amino acids.

(3) $$\mathrm{CPS}=\mathrm{Ln}\left(\frac{f\left(\mathrm{AB}\right)}{f\left(A\right)\times f\left(B\right)}\times \frac{f\left(X\right)\times f\left(Y\right)}{f\left(\mathrm{XY}\right)}\right)$$

(4) $$\mathrm{CPB}=\frac{\sum_{i}^{l}\mathrm{CP}S_{i}}{l-1}$$

### Calculation of the CAI

The codon adaptation index was calculated with the Emboss program [49] based on the frequency of codon usage of *Salmo salar*.

### Prediction of free folding energy

Free folding energy was calculated with the Mfold program through the web server (http://mfold.rna.albany.edu/?q=mfold) using the standard parameters. The energy was calculated for the region between residuals 330 and 371 with respect to HE SK779 HPR0.

### Virulence data

The virulence of 11 ISA viruses with known genomes was obtained from data published by Mjaaland et al. in 2005, in which they reported mortality rates 60 days after infection. This work analyzed the mortality rates of two fish populations from the same parents and the mortality rates of fish with genetic heterogeneity. The fish with the same parents were infected by cohabitation (TGI and TGII) with genetically heterogeneous fish that were previously infected by intraperitoneal injection (COH) [36].

### HE genes of ISAV

The complete sequence of the codifying regions of the ISAV HE genes was downloaded from Genbank. All the genes that did not begin with ATG were filtered out, ending with a stop codon and ambiguous nucleotides. A total of 63 sequences passed through the filter. The GenBank codes of these sequences can be found in the supplementary material.

### Graphs and statistical methods

The graphs and statistical tests were made using the program SigmaPlot 11. The significance of the differences or correlations among the data groups obtained was evaluated with non-parametric tests (Rank Sum Test for comparing two groups or Spearman Rank Order for correlations) using a value of p < 0.05 as a cutoff.

## Competing interests

The authors declare no competing interests.

## Authors’ contributions

MT designed all *in silico* experiments. JMS performed all computational analysis and calculations. ES contributed to data analysis and preparing the manuscript. All authors read and approved the final manuscript.

## Supplementary Material

Additional file 1 **This is a Microsoft Word document containing supplementary figures about Gibb’s free energy of folding acquired by different HPRs, Pattern of codon-pair usage in *Salmo salar, *Pattern of distribution of the bias of codon-pair use in *S. Salar, *CPB values of HE genes, CPB values according to the HPR type, Effect of the HPR region on bias in the use of codon pairs, and CPB values of the HPR regions, and W values of codons present in the HPR0 region of HE gene.** Click here for file

Additional file 2 **This is a Microsoft Excel document containing a supplementary table of *Salmo salar *tRNA copy numbers.** Click here for file

Additional file 3 This is a Microsoft Excel document of a supplementary table containing HE gene ID, HE Protein ID, HPR or group, Country of isolation, HE Protein size, CPB value of HE, CPB of HPR, Name of ISAV strain and mortality rates of different ISAV isolates. Click here for file

Additional file 4 **This is a Microsoft Excel document containing a supplementary table with tRNA availability (W values) for each coding codon of *Salmo salar.*** Click here for file

Additional file 5 This is a Microsoft Excel document containing a supplementary table with tRNA availability (W values) for each codon present in HPR0. Click here for file

Additional file 6 **This is a Microsoft Excel document containing a supplementary table with the Genebank access numbers for *Salmo salar *genes analyzed in this work.** Click here for file

Additional file 7 **This is a Microsoft Excel document containing a supplementary table with CPS values for *Salmo salar.*** Click here for file
