# Supplementary material for: Analysis of the use of codon pairs in the HE gene of the ISA virus shows a correlation between bias in HPR codon-pair use and mortality rates caused by the virus
Source: Virol J. 2013 Jun 6;10:180. doi: 10.1186/1743-422X-10-180 (PMC3684539; doi:10.1186/1743-422X-10-180)
Supplement: Additional file 1 — This is a Microsoft Word document containing supplementary figures about Gibb’s free energy of folding acquired by different HPRs, Pattern of codon-pair usage in Salmo salar, Pattern of distribution of the bias of codon-pair use in S. Salar, CPB values of HE genes, CPB values according to the HPR type, Effect of the HPR region on bias in the use of codon pairs, and CPB values of the HPR regions, and W values of codons present in the HPR0 region of HE gene. [file 1743-422X-10-180-S1.doc]

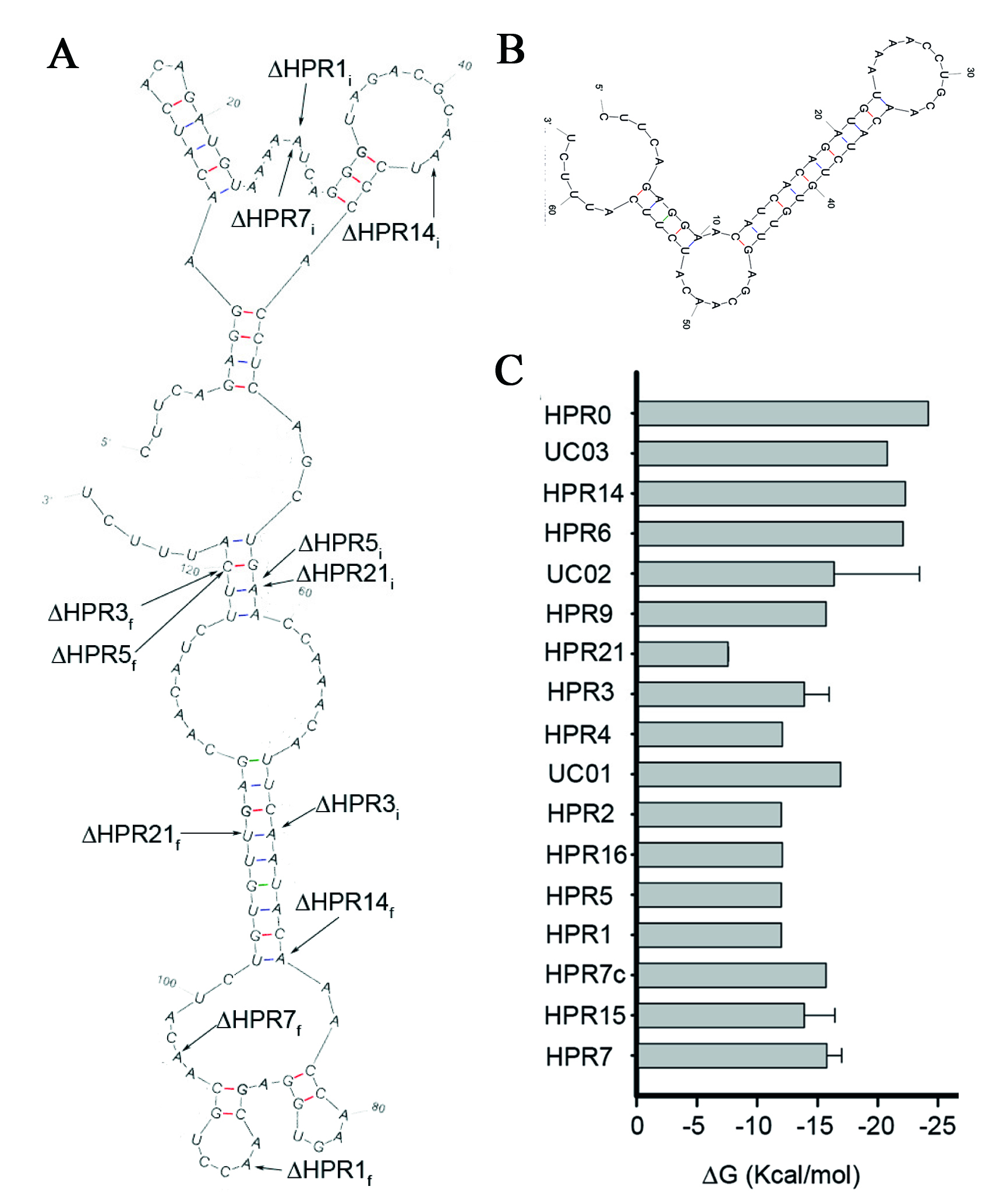


**Supplementary figure 1: Gibb’s free energy of folding acquired by different HPRs –** The figure shows the secondary structure of HPR0 region (Panel A) and the position of the selected zones in the different HPRs. The region between ΔHPR(X)_i_ and ΔHPR(X)_f_ correspond to the region deleted in the HE gene with the HPR(X) region. Panel B show the secondary structure of HPR1. The figure also shows the Gibb’s free energy value associated with the secondary structure pre-identified by the Mfold program (Panel C).


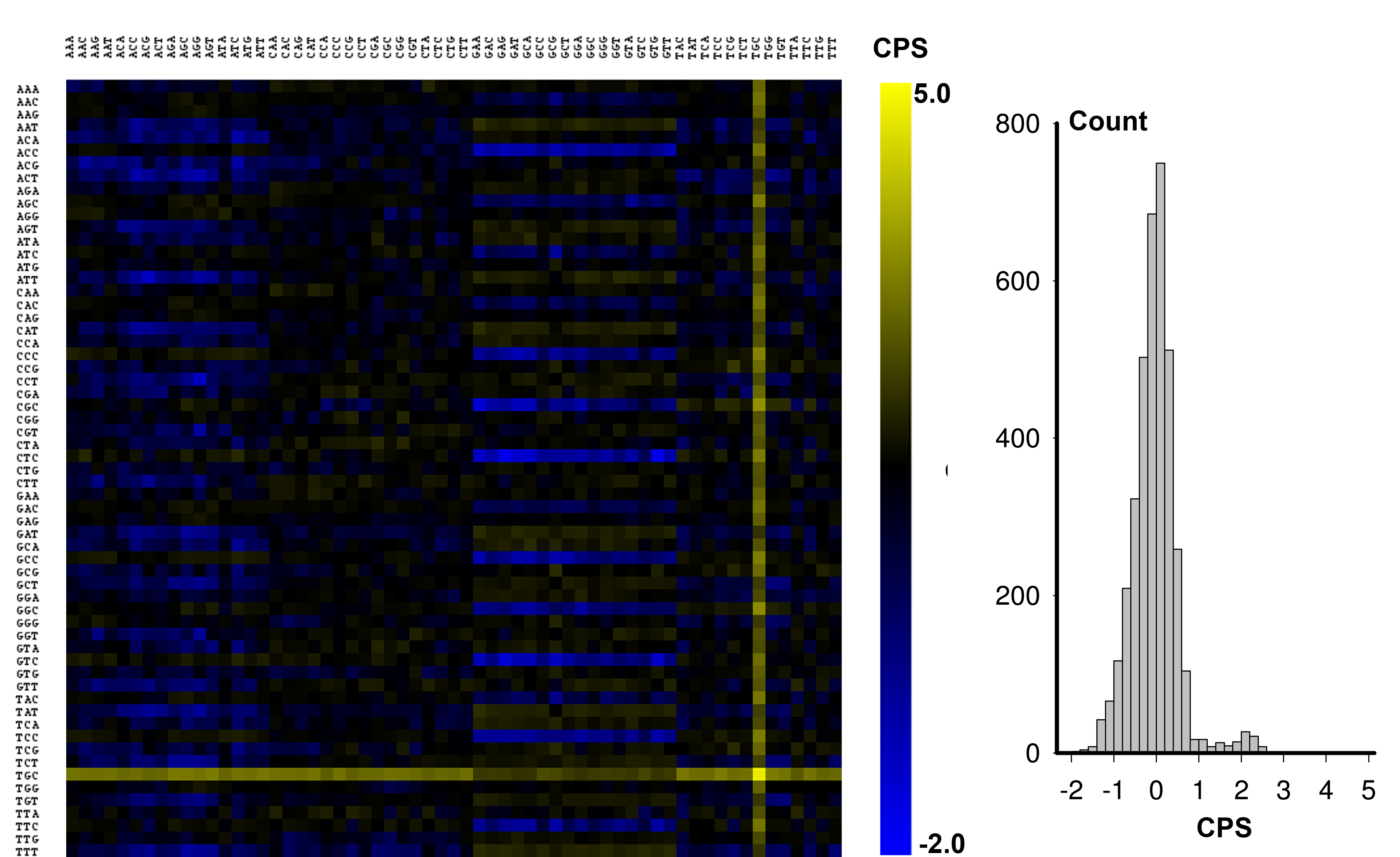


**Supplementary figure 2: Pattern of codon-pair usage in *Salmo salar*** – The left panel shows CPS values of the 61x61 combinations of codon pairs in 3489 cds. The right panel shows the histogram of the distribution of CPS values.


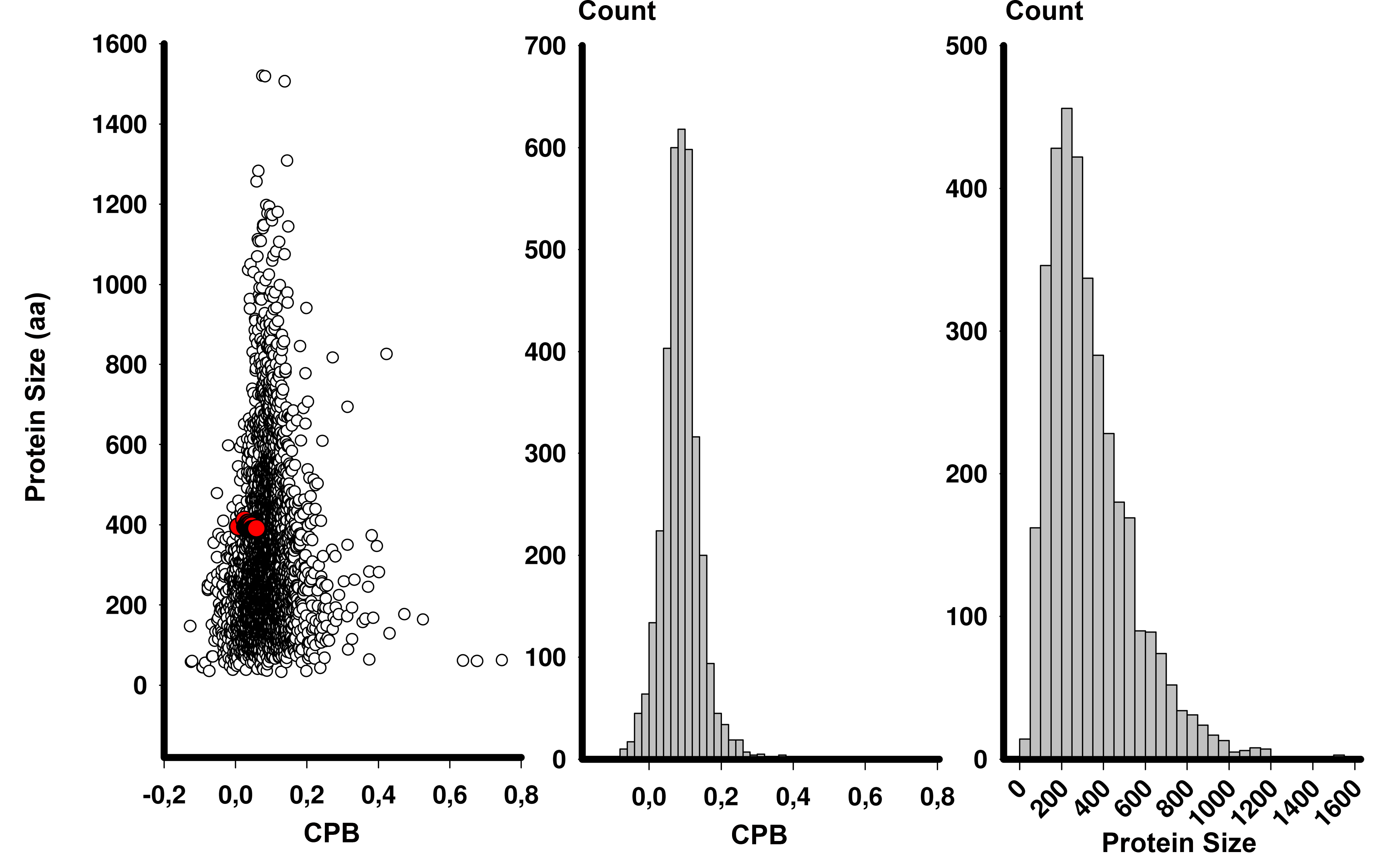


**Supplementary figure 3: Pattern of distribution of the bias of codon-pair use in *S. Salar* –** The left panel shows the distribution of the CPB values of the *Salmo salar* genes according to the size of the protein that they codify. The CPB values of the ISAV HE genes are shown in red. The center panel shows the distribution of CPB values in the Salmo salar genes. The right panel shows the size distribution of the codified proteins in the analyzed *Salmo salar* genes.

*

**Supplementary figure 4**: **CPB values of the HE genes –** The figure shows the mean CPB values of the HE genes classified according to the country of origin. * p = 0.008, t-test. Chilean viruses n= 32, Norwegian viruses n= 21, Canadian viruses n = 5, UK viruses n = 5

**Supplementary figure 5: CPB values according to the HPR type –** The figure shows the CPB value of the HE gene according to the HPR type present.

**Supplementary figure 6: Effect of the HPR region on bias in the use of codon pairs –** The figure shows the contribution of the HPR region on the CPB value of the HE gene. QCPB values (CPB/(CPQ-H) higher than 1 imply that the HPR region increases the CBP value of the HE gene. QCPB values of less than 1 imply that the HPR region negatively affects the CPB value of the HE gene. CPB corresponds to the CPB value using all the codons of the gene. CPB-H corresponds to the CPB value of the gene without the HE region, corresponding to HE genes with HPR without classification.

**Supplementary figure 7: CPB value of the HPR region –** The figure shows the CPB values of the analyzed HPR regions. HPR0 is show in a black bar.

**Supplementary figure 8**: **W values of codons present in the HPR0 region of HE gene**. The figure shows the W values of each codon between residues 229 and 270 from HE gene and encode for the HPR0. W values were calculated in relation to the tRNA gene copy number. Three regions with decrease values of adaptation to the tRNA pool are signalized with red box.
